# Commentary: Awareness of Risk Factors for Digital Game Addiction: Interviewing Players and Counselors

**DOI:** 10.3389/fpsyg.2015.01913

**Published:** 2015-12-16

**Authors:** Gilbert E. Franco

**Affiliations:** Graduate Behavioral Sciences, Southern California SeminaryEl Cajon, CA, USA

**Keywords:** videogames, addiction, addiction research, counseling psychology, digital games

The videogame industry is a multibillion dollar industry. In 2009, consumers spent more than 22.41 billion US dollars in videogame related merchandise (Entertainment Software Association, 2015). Perhaps as a result of its success and media attention, studies on videogame playing have increased in recent years (e.g., Gentile et al., 2004; Anand, 2007; Holtz and Appel, 2011; Kahlbaugh et al., 2011; Watson et al., 2011). One such study, *Awareness of risk factors for digital game addiction: Interviewing players and counselors*, looks at the risk factors of digital game addiction from both a player and a counselor perspective (Kneer et al., 2014).

Kneer et al. (2014) conducted a study to evaluate perceived digital game risk factors. The perceptions of 28 non-addicted videogame players and 7 counselors were recorded using individual interviews. It was found that digital game players identified social settings as an important factor associated with problematic digital game playing. It was also found that counselors looked at existing psychological problems.

Based on their findings, Kneer et al. (2014) argue that the experiences of both the counselor and videogame playing groups should be taken into account when developing prevention and intervention programs. In addition, the experiences of friends, family, and parents of videogame players should be taken into consideration. The experiences of friends, family, and parents may give a more complete picture on the etiology of problematic videogame playing. In other words, a parent can have insights that can add to the efficacy of a child's treatment for problematic videogame behaviors.

If a child is playing first person shooters online for 5 h a day and her grades in school are dropping, a parent's perspective can help determine a client's course of treatment. In one scenario, a parent can say that the client locks herself in her room and plays for 5 h a day by herself. In another scenario, a parent can say that she works until 8:00 p.m. each weeknight and did not know prior to session that her child was playing for 5 h a day and having trouble in school. A therapist or counselor can take different approaches to treatment based on what input he or she gets from a client and their significant others.

Nikken and Jansz (2006) researched the mediation strategies that a parent would use to regulate their child's videogame playing. They conducted an internet survey of 536 parent-child dyads and found that both parents and children had congruent views on the application mediation strategies for video game use (Nikken and Jansz, 2006). Parents and children having congruent views can have implications for prevention and intervention programs through the use of family therapy or behavioral health assessments that include the client's family.

Kneer et al. (2014) suggest that the experience of videogame players should be taken into consideration when developing an assessment for videogame addiction because their knowledge about videogames and videogame addiction comes from experience and exposure to it. In contrast, they argue that counselors obtain their knowledge and views about videogames and videogame addiction through their disciplinary knowledge. In addition to using the player and counselor perspectives in developing videogame addiction assessments, developing an assessment using the perspective of parents and family members can give an evaluator a more systemic perspective as to the etiology of videogame addiction.

Kneer et al. (2014) bring up relevant points in the development of prevention and early intervention programs and in the development of assessments for videogame addiction. Both videogame players and counselors can bring their own perspectives into the development of prevention programs and assessments. In addition to these perspectives, it is suggested that additional research on the effects of videogame addiction on the parents and family of the videogame player can bring a more systemic perspective on videogame addiction.

The systemic approach is holistic in scope and focuses on reciprocal causality of challenges that a client experiences (Bateson, 1972; Becvar and Becvar, 2003). Recent studies have been conducted on multifamily group therapy and internet addiction (i.e., Liu et al., 2015) and brief strategic family therapy and parent and child substance use (i.e., Horigian et al., 2015). For example, Horigian et al. (2015) found that brief strategic family therapy was effective in reducing parent and adolescent alcohol use. Additionally, Liu et al. (2015) found that multifamily group therapy was effective in reducing adolescent internet addiction. On the other hand, there is a paucity of research available in family therapy and videogame addiction.

By looking at videogame addiction through a systemic perspective and conducting research on the parents and family members of video game players, perhaps we can gain a better understanding of this form of addiction. Conducting more research in the effectiveness of family therapy in treating videogame addiction can, in addition to adding to our understanding of videogame addiction, can potentially provide clients more effective treatment options. If family therapy is as effective in treating videogame addiction as it is in treating alcohol abuse, then client quality of care can improve as a result.

## Conflict of interest statement

The author declares that the research was conducted in the absence of any commercial or financial relationships that could be construed as a potential conflict of interest.
